# Ageing Effects, Generation Means, and Path Coefficient Analyses on High Kernel Elongation in Mahsuri Mutan and Basmati 370 Rice Populations

**DOI:** 10.1155/2021/8350136

**Published:** 2021-05-21

**Authors:** Anna Arina Bt Ab. Halim, Mohd Y. Rafii, Mohamad B. Osman, Yusuff Oladosu, Samuel C. Chukwu

**Affiliations:** ^1^Department of Crop Science, Faculty of Agriculture, Universiti Putra Malaysia (UPM), 43400 Serdang, Selangor, Malaysia; ^2^Institute of Tropical Agriculture and Food Security, Universiti Putra Malaysia (UPM), 43400 Serdang, Selangor, Malaysia; ^3^Department of Crop Production and Landscape Management, Faculty of Agriculture and Natural Resources Management, Ebonyi State University, Abakaliki, Nigeria

## Abstract

High kernel elongation (HKE) is one of the high-quality characteristics in rice. The objectives of this study were to determine the effects of ageing treatments, gene actions, and inheritance pattern of kernel elongation on cooking quality in two populations of rice and determine the path of influence and contribution of other traits to kernel elongation in rice. Two rice populations derived from crosses between MR219 × Mahsuri Mutan and MR219 × Basmati 370 were used. The breeding materials included two F_1_ progenies from the two populations, and their respective parents were grown in four different batches at a week interval to synchronize the flowering between the female and male plants. Scaling tests and generation means analysis were carried out to determine ageing effects and estimate additive-dominance gene action and epistasis. The estimation of gene interaction was based on quantitative traits. Path coefficient analysis was done using SAS software version 9.4 to determine the path of influence (direct or indirect) of six quantitative traits on HKE. Results obtained showed that nonallelic gene interaction was observed in all traits. The results before ageing and after ageing showed significant differences in all traits, while the gene interaction changed after ageing. The HKE value improved after ageing, suggesting that ageing is an external factor that could influence gene expression. The epistasis effect for HKE obtained from the cross Mahsuri Mutan × MR219 showed duplicate epistasis while that obtained from a cross between Basmati 370 × MR219 showed complimentary epistasis. Besides, the heritability of HKE was higher in Basmati 370 × MR219 compared to that obtained in Mahsuri Mutan × MR219. The path analysis showed that the cooked grain length and length-width ratio positively significantly affected HKE. It was concluded that ageing treatment is an external factor that could improve the expression of HKE. The findings from this study would be useful to breeders in the selection and development of new specialty (HKE) rice varieties.

## 1. Introduction

Rice (*Oryza sativa* L.) is a major food in the world, especially in Asia [1, 2]. Rice is the only cereal crop cooked and consumed mainly as whole grain, and quality considerations are more important [3]. Quality rice becomes a demand in the selection of rice, and it is increasing day by day. High-quality rice can influence the grading and price of rice in the market. Moreover, quality characteristics can increase the total economic value of rice. Therefore, improving rice grain quality has been the main objective in starting a breeding programme to meet consumer preferences and market demand. Generally, rice quality traits include milling quality, appearance quality, and nutritional quality based on cooking and eating values. Most rice consumers will value the rice quality based on these categories. Therefore, selection for improved cooking and eating qualities is important to fulfill the consumer needs. Physical quality properties such as size, shape, uniformity, and general appearance (kernel shape and *L*/*B* ratio are important features while assessing grain quality [4, 5]). The gelatinization temperature (GT), gel consistency (GC), and amylose content (AC) are directly related to cooking and eating quality [5]. Starch (amylose and amylopectin) and protein composition are equally important in determining the cooking quality of rice [3, 5]. The rice grain quality varies from one geographical region to another and depends on consumer preference [6]. For example, in *japonica* rice-eating countries, low amylose and short grains are preferred, while in *indica* rice-consuming countries, long grains with intermediate amylose and alkali spreading value, soft gel consistency, and high-volume expansion of cooked rice are preferred [7].

Studies on genetic variability of quality rice have been widely reported. The information on genetic improvement is very crucial in improving high-quality rice. Genetic improvement depends on the effectiveness of the selection of progenies with different genetic values. Generation means analysis has been used to estimate the gene actions controlling quantitative traits such as additive-dominance effects, and their interactions are known as the gene action which is associated with breeding value [8]. The additive-dominance model, which is the mean value of the generation, can identify the additive and dominance effect of different traits [9]. The model could explain the inheritance in terms of additive and dominance properties of the single gene different from the phenotypic characteristics. Generation means analysis consists of six generations which are parent 1 (P_1_), parent 2 (P_2_), first progeny (F_1_), second generation (F_2_), first progeny backcrossed with recurrent parent 1 (BC_1_P_1_), and first progeny backcrossed with recurrent parent 2 (BC_1_P_2_). These generations were used to estimate the gene action and linkage. Ramli et al. [10] used these types of analyses to demonstrate the grain quality traits under selected rice populations derived from different amylose characteristics. There is a limited study of gene action through generation means analysis in improving Malaysia rice variety. Therefore, the gene action and heritability of the grain and cooked rice information were important for selection in the breeding programme. Thus, the study was carried out to estimate the additive, dominance, and epistasis effects and heritability, to determine the inheritance pattern of kernel elongation for cooking quality in the two cross generations, and to determine the path of influence and the contribution of other traits to kernel elongation.

## 2. Materials and Methods

### 2.1. Population Development

This study used three rice varieties, including two high kernel elongation rice varieties, Mahsuri Mutan and Basmati 370, and normal kernel elongation rice variety MR219. Mahsuri Mutan and Basmati 370 rice varieties served as donor parents and were crossed with MR219. The seed was germinated and transferred to the pot in four batches at a week interval to synchronize flowering between female and male plants. The healthy plants were selected for the crossing. Two F_1_ generations were developed from crossing between MR219 × Mahsuri Mutan and MR219 × Basmati 370. All breeding materials, including the F_1_ progenies from the two populations and their respective parents, were grown in four different batches at a week interval to synchronize the flowering between the female and male plants. The F_1_ progenies of MR219 × Mahsuri Mutan were backcrossed with their respective parents to develop BC_1_P_MR219_ and BC_1_P_MM_. Similarly, the F_1_ progenies of MR219 × Basmati 370 were backcrossed to develop BC_1_P_MR219_ and BC_1_P_B370_. Concurrently, during the development of the backcross populations, the parents (Mahsuri Mutan, Basmati 370, and MR219) and F_1_ progenies of both crosses were selfed for true seeds and F_2_ seeds, respectively. The seeds of all populations were harvested at the maturity stage according to each population. The population sizes for each cross were followed as described by Ramli et al. [10].

### 2.2. Experimental Design, Crop Maintenance, and Harvesting

All breeding populations including the parental varieties of Mahsuri Mutan- and Basmati 370-based populations were evaluated in two separate experimental areas in the glasshouse. For each population, the sample size was 20 plants for each parent, 60 plants for F_1_, 160 plants for F_2_, and 120 each for BCP_1_ and BCP_2_ generations. The plants were grown in a pot with three plants per pot and arranged according to generation rows. The fertilizer NPK granulated was applied in three split applications. The pot's water level was maintained at 7-10 cm from transplanting to 70 days and was slowly drained out for harvesting. Pests and diseases were monitored appropriately and will be treated when the need arises. The samples were harvested at the maximum maturity stage for each generation. All plant samples were individually harvested and tagged. The plants were dried under sunlight before manual threshing. The threshed grains were dried in an oven at 40°C overnight (Memmert, Germany). The dried grains from each generation were dehusked using a dehusker motion Smith machine and rice testing milling machine (BS08A Satake Co., Ltd., Tokyo, Japan).

### 2.3. Ageing and Nonageing Treatments

Only unbroken rice kernels were used for the kernel elongation ratio measurement. For artificial ageing treatment, the samples were heated at 90°C in the oven (Memmert, Germany) for 3 hours. After the heat treatment, the samples were cooled at room temperature (25°C) for 1 hour according to the method described by Faruq et al. [11]. Then, the samples were cooked for the determination of the kernel elongation ratio.

### 2.4. Data Collection and Statistical Analysis

Data on the grain physical properties were measured according to the rice descriptor [12]. Physical properties measured included milled grain length (MGL), cooked grain length (CGL), milled grain width (MGW), cooked grain width (CGW), length-width ratio (LW), and high kernel elongation ratio (HKE). The elongation ratio was determined using the following equation:

(1) $$\begin{array}{c} \text{Elongation ratio}=\frac{\text{Average of cooked kernels}}{\text{Average length of raw kernels}}. \end{array}$$

The proportionate change (PC) of kernel elongation before and after cooking was measured using the following equation:

(2) $$\begin{array}{c} \text{PC}=\frac{L_{\text{F}}/B_{\text{F}}-L_{0}/B_{0}}{L_{0}/B_{0}}, \end{array}$$

where PC is the proportionate change, *L*_F_ and *B*_F_ are the length and breadth of the kernel after being cooked, respectively, and *L*_0_ and *B*_0_ are the length and breadth of the kernel after being cooked.

### 2.5. Scaling Test

The mean kernel elongation of rice for every generation of each cross was used to determine the gene effect and heritability for both crosses. The most important condition in analyzing gene effects is assessing the adequacy of the additive-dominance model using a scaling test. Three scales, i.e., *A*, *B*, and *C*, were estimated following the equation derived from Singh and Chaudhary [13].

The respective variance was calculated according to the following equation:

(3) $$\begin{array}{c} V_{A}=4V\left({\text{BC}}_{1}\right)+V\left({\text{P}}_{1}\right)+V\left({\text{F}}_{1}\right),\\ V_{B}=4V\left({\text{BC}}_{2}\right)+V\left({\text{P}}_{2}\right)+V\left({\text{F}}_{1}\right),\\ V_{C}=16V\left({\text{F}}_{2}\right)+4V\left({\text{F}}_{1}\right)+V\left({\text{P}}_{1}\right)+V\left({\text{P}}_{1}\right), \end{array}$$

where P_1_ is parent 1, P_2_ is parent 2, F_1_ is the progeny derived, BCP_1_ is the backcross of the recurrent parent (P_1_) to the respective F_1_, BCP_2_ is the backcross of recurrent parent (P_2_) to respective F_2_, *V*(P_1_) is the variance of parent 1, *V*(P_2_)is the variance of parent 2, *V*(F_1_)is the variance of the derived progeny, *V*(BC_1_)is the variance of the backcross of the recurrent parent (P_1_) with the respective F_1_, and *V*(BC_2_) is the variance of the backcross of the recurrent parent (P_2_) with the respective F_1_. The standard error (SE) was determined from the square root of the respective variance, and Student's *t*-test was performed by dividing the value by SE: SE(*A*) = (*V*_*A*_) 1/2 and *t*(*A*) = *A*/SE(*A*), where *V*_*A*_ is the variance of scale *A*. A similar procedure was adopted to determine the standard errors for scales *B* and *C* and their respective *t* values. The calculated value of *t* was compared with the tabulated value at 5% level of significance. The significance of any of the scales indicated the presence of nonallelic interactions. The nonsignificance of all scales indicated the absence of nonallelic interaction. Generally, the adequacy of an additive-dominance model later suggested the estimation of gene effects using the three-parameter model, whereas the nonadequacy of the model suggested the use of the six-parameter model.

### 2.6. Components of Generation Means

There are two types of models involved in determining gene effects: the three-parameter model that has been used in the absence of nonallelic interactions and the six-parameter model that has been used in the presence of nonallelic interactions or called as the joint scaling model [10, 14, 15]. In the absence of epistasis interactions, which are equivalent to adherence to the additive-dominance model, the mean (*m*), additive effect (*d*), and dominance effect (*h*) were determined according to the following equations as described by Parihar et al. [16]:

(4) $$\begin{array}{c} \left[m\right]=\frac{1}{2}{\text{P}}_{1}+\frac{1}{2}{\text{P}}_{2}+4{\text{F}}_{2}-2\text{B}{\text{C}}_{1}-2\text{B}{\text{C}}_{2},\\ \left[d\right]=\frac{1}{2}{\text{P}}_{1}-\frac{1}{2}{\text{P}}_{2},\\ \left[h\right]=6\text{B}{\text{C}}_{1}+6\text{B}{\text{C}}_{2}-8{\text{F}}_{2}-{\text{F}}_{1}-1.5{\text{P}}_{1}-1.5{\text{P}}_{2}, \end{array}$$

where [*m*] is the mean epistasis effect, [*d*] is the additive effect, and [*h*] is the dominance effect. Their variances were computed using the following formulae:

(5) $$\begin{array}{c} V_{m}=\frac{1}{4}V\left({\text{P}}_{1}\right)+\frac{1}{4}V\left({\text{P}}_{2}\right)+16\;V\left({\text{F}}_{2}\right)+4V\left({\text{BC}}_{1}\right)+4V\left({\text{BC}}_{2}\right),\\ V_{d}=\frac{1}{4}V\left({\text{P}}_{1}\right)+\frac{1}{4}V\left({\text{P}}_{2}\right),\\ V_{h}=36V\left({\text{BC}}_{1}\right)+36V\left({\text{BC}}_{2}\right)+64V\left({\text{F}}_{2}\right)+V\left({\text{F}}_{1}\right)+\frac{9}{4}V\left({\text{P}}_{1}\right)+\frac{9}{4}\left({\text{P}}_{2}\right), \end{array}$$

where *V*_*m*_ is the mean variance, *V*_*d*_ is the additive variance, *V*_*h*_ is the dominance variance, *V*(P_1_) is the variance of parent 1, *V*(P_2_) is the variance of parent 2, *V*(F_1_) is the variance of the progeny derived, *V*(BC_1_) is the variance of the backcross of the recurrent parent (P_1_) with the respective F_1_, and *V*(BC_2_) is the variance of the backcross of recurrent parent (P_2_) with the respective F_1_. The standard error (SE) was determined as the square root of the respective variance, and Student's *t*-test was performed by dividing the value of the effect by the SE of the effect.

(6) $$\begin{array}{c} {\text{SE}}_{\left(m\right)}={\left(V_{m}\right)}^{1/2},\\ t_{\left(m\right)}=\frac{m}{{\text{SE}}_{\left(m\right)}}. \end{array}$$

The calculated value of “*t*” was compared with the tabulated values at the 5% level of significance. In the case of the presence of nonallelic interactions or an epistasis effect, the interactions were calculated separately. The respective gene effect was calculated using the mean value of the respective generation using the following equations described by Ramli et al. [10], Hayman [15], and Khatun et al. [17]:

(7) $$\begin{array}{c} \left[m\right]=\text{mean of }{\text{F}}_{2},\\ \left[d\right]={\text{BC}}_{1}-{\text{BC}}_{2},\\ \left[h\right]={\text{F}}_{1}-4\;{\text{F}}_{2}-\frac{1}{2}{\text{P}}_{1}-\frac{1}{2}{\text{P}}_{2}+2\text{B}{\text{C}}_{1}+2\text{B}{\text{C}}_{2},\\ \left[i\right]=2\text{B}{\text{C}}_{1}+2\text{B}{\text{C}}_{2}-4{\text{F}}_{2},\\ \left[j\right]=2\text{B}{\text{C}}_{1}-{\text{P}}_{1}-2\text{B}{\text{C}}_{2}+{\text{P}}_{2},\\ \left[l\right]={\text{P}}_{1}+{\text{P}}_{2}+2{\text{F}}_{1}+4{\text{F}}_{2}-4\text{B}{\text{C}}_{1}-4\text{B}{\text{C}}_{2}, \end{array}$$

where [*m*] is the mean, [*d*] is the additive gene effect, [*h*] is the dominance gene effect, [*i*] is the interaction of the additive × additive gene effect, [*j*] is the interaction of the additive × dominance gene effect, and [*l*] is the interaction of the dominance × dominance gene effect. The variance of the respective gene effects and their interactions was calculated using the following equations:

(8) $$\begin{array}{c} V_{m}=V\left({\text{F}}_{2}\right),\\ V_{d}=V\left({\text{BC}}_{1}\right)+V\left({\text{BC}}_{2}\right),\\ V_{h}=V\left({\text{F}}_{1}\right)+4\text{F}\left({\text{F}}_{2}\right)+\frac{1}{2}V\left({\text{P}}_{1}\right)+\frac{1}{2}V\left({\text{P}}_{2}\right)+2V\left({\text{BC}}_{1}\right)+2V\left({\text{BC}}_{2}\right)+2V\left({\text{BC}}_{2}\right),\\ V_{i}=2V\left({\text{BC}}_{1}\right)+2V\left({\text{BC}}_{2}\right)+4\left({\text{F}}_{2}\right),\\ V_{j}=2V\left({\text{BC}}_{1}\right)+V\left({\text{P}}_{1}\right)+2V\left({\text{BC}}_{2}\right)+V\left({\text{P}}_{2}\right),\\ V_{i}=V\left({\text{P}}_{1}\right)+V\left({\text{P}}_{2}\right)+2V\left({\text{F}}_{1}\right)+4V\left({\text{F}}_{2}\right)+4V\left({\text{BC}}_{1}\right)+4V\left({\text{BC}}_{2}\right), \end{array}$$

where *V*_*m*_ is the mean variance, *V*_*d*_ is the additive variance, *V*_*h*_ is the dominance variance, *V*_*i*_ is the variance for the interaction of additive × additive gene effects, *V*_*j*_ is the variance for the interaction of additive × dominance gene effects, *V*_*l*_ is the variance for the interaction of dominance × dominance gene effects, *V*(P_1_) is the variance of parent 1, *V*(P_2_) is the variance of parent 2, *V*(F_1_) is the variance of the progeny derived, *V*(BC_1_) is the variance of the backcross of the recurrent parent (P_1_) with the respective F_1_, and *V*(BC_2_) is the variance of the backcross of recurrent parent (P_2_) with the respective F_1_.

### 2.7. Estimation of Heritability

Broad sense (*h*^2^_b_) and narrow sense (*h*^2^_n_) heritability for the F_2_ populations were estimated based on the ratio of total genetic variation to total phenotypic variation:

(9) $$\begin{array}{c} {h^{2}}_{\text{b}}=\frac{V{\text{F}}_{2}-\left[\left(V{\text{P}}_{1}+V{\text{P}}_{2}+2V{\text{F}}_{1}\right)/4\right]}{V{\text{F}}_{2}},\\ {h^{2}}_{\text{n}}=\frac{V{\text{F}}_{2}-\left[\left({\text{BCP}}_{1}+V{\text{BP}}_{2}\right)/2\right]/2}{V{\text{F}}_{2}}. \end{array}$$

### 2.8. Inheritance Pattern

Kernel elongation ratio data of F_2_ generations were used to show the inheritance pattern, which followed the Mendelian inheritance pattern 3 : 1 (nonelongation : elongation). The *χ*^2^ test was done on F_2_ generation to determine the inheritance pattern [9]. All quantitative data were analyzed following the analysis of variance (ANOVA) procedure using SAS software version 9.4. Differences were declared statistically significant at *P* < 0.05. The means were separated by the least significant difference (LSD) at 5% probability level where significant differences were detected. Interrelationships among trait values were estimated using the Pearson correlation coefficient.

### 2.9. Phenotypic Path Coefficient Analysis in High Kernel Elongation of Rice

Data were collected from grain quality characters. A total of six dependent variables were recorded. The quality of milled and cooked rice was recorded to find the linkage to the effect of HKE on cooking rice. The milled grain length, cooked grain length, milled grain width, cooked grain width, proportionate change, ratio of milled grain length-width and ratio of cooked grain length-width, and high kernel elongation ratio of the rice parameters were analyzed. The phenotypic correlation was further partitioned into direct and indirect components using path coefficient analysis following Oladosu et al. [18], decomposed with SAS software version 9.4. The path coefficients were obtained by working out sets of simultaneous equations arranged in matrix notation, which showed the relationships between correlations and path coefficients as described by Usman et al. [19].

Effect of grain quality parameters on the HKE per plant:

(10) $$\begin{array}{c} r_{17}=P_{17}+r_{12}P_{27}+r_{13}P_{37}+r_{14}P_{47}+r_{15}P_{57}+r_{16}P_{67},\\ r_{27}=r_{21}P_{17}+P_{27}+r_{23}P_{37}+r_{24}P_{47}+r_{25}P_{57}+r_{26}P_{67},\\ r_{37}=r_{31}P_{17}+r_{32}P_{27}+P_{37}+r_{34}P_{47}+r_{35}P_{57}+r_{36}P_{67},\\ r_{47}=r_{41}P_{17}+r_{42}P_{27}+r_{43}P_{47}+P_{47}+r_{45}P_{57}+r_{46}P_{67},\\ r_{57}=r_{51}P_{17}+r_{52}P_{27}+r_{53}P_{37}+r_{54}P_{47}+P_{57}+r_{56}P_{67},\\ r_{67}=r_{61}P_{17}+r_{62}P_{27}+r_{63}P_{37}+r_{64}P_{47}+r_{65}P_{57}+P_{67}, \end{array}$$

where the character arrangement is shown as follows: 1—milled grain length (MGL), 2—cooked grain length (CGL), 3—milled grain width (MGW), 4—cooked grain width (CGW), 5—proportionate change (PC), 6—length-width ratio (LW), and 7—high kernel elongation (HKE).

In the above equations, *r* values are the phenotypic correlations between variables, *P* values are the direct effects (coefficients) of one variable upon another, and *r*_*ij*_*P*_*ij*_ values are the indirect effects.

## 3. Results and Discussion

The overall ANOVA for the generation of the entire population was summarized and is presented in Table 1. These populations were derived from the crosses between MR219 × Mahsuri Mutan and MR219 × Basmati 370, which have characteristics of high kernel elongation (Mahsuri Mutan and Basmati 370) and normal kernel elongation (MR219) after cooking. The six generations consist of the parents, first filial progeny (F_1_), second generation (F_2_), and the backcross of F_1_ with their respective recurrent parents (BC_1_P_1_ and BC_1_P_2_). Highly significant differences were observed for both populations derived from MR219 × Mahsuri Mutan and MR219 × Basmati 370 for all evaluated traits. Similarly, significant differences were observed between ageing treatments for all generations except for the length-width ratio (LW) where a nonsignificant difference was observed. Changes in rice grain character when cultivated in a diverse environment have been reported [6, 20]. The analysis showed that ageing directly affected the different results on all parameters across the generation. According to Faruq et al. [21], rice showed higher kernel elongation after ageing treatment. Besides, ageing is a process that improves rice cooking quality and helps in increasing the elongation rate upon cooking. They also proved that ageing could significantly influence the kernel elongation ratio and other related traits. Faruq et al. [21] concluded from his study on the inheritance of kernel elongation that the kernel elongation gene is tightly linked with cooked elongation. In this study, the result of proportionate change and high kernel elongation showed a significant difference, which revealed that cooking may affect the expression of high kernel elongation.

The results obtained from this study indicated that crosses between Basmati 370 × MR219 showed higher value of HKE than crosses of Mahsuri Mutan × MR219. This showed that the performance of the cross involving Mahsuri Mutan was lower than that of Basmati 370. Basmati 370 showed more dominance on high kernel elongation traits due to their origin. This can be related to the origin of Basmati variety itself. Based on the studies conducted by Sood and Siddiq [22] on the geographical distribution of kernel elongation gene(s) in rice, varieties showing high kernel elongation on cooking were found to be traditionally cultivated in the northwest part of undivided India. Thus, it can explain the expression of high kernel elongation in Basmati stronger than Mahsuri Mutan due to the gene origin, contrary to Mahsuri Mutan where the high kernel elongation could only be expressed after ageing treatment. Moreover, Mahsuri Mutan originated from Mahsuri variety, a local variety that only expresses normal elongation after cooking. Thus, the gene expression in Mahsuri Mutan must be induced by an external factor such as ageing. According to Mohamad [23], Mahsuri Mutan was the only local variety that has a high kernel elongation gene. This mutant was found to improve cooking and eating qualities, possessing an elongation characteristic similar to that found in Basmati. This highly prized attribute should be used in plant breeding programmes to produce high-quality local rice, especially with high kernel elongation traits.

### 3.1. Generation Means of the Population Derived from MR219 × Mahsuri Mutan

The average performance of each generation is presented in Table 2. In general, the average mean of F_1_ for all traits is lower than the mean of parent Mahsuri Mutan except for MGW and CGW. For F_2_, all traits were slightly lower than those for the F_1_ generation except for PC. When the F_1_ was backcrossed to its recurrent parent, the average performance of the BC_1_P_1_ generation was higher than that of F_1_ for most of the traits except MGW, CGW, and LW. The average performance of BCP_1_ was also higher compared with that of the second backcross (BC_1_P_2_) for MGL, CGL, HKE, PC, and LW except MGW and CGW which had slightly higher values. This finding revealed that P_1_ plants were the superior parent. Mean separation by means of LSD revealed the differences between the generations for the grain quality traits evaluated (Table 2). Grain physical properties for CGL had no significant differences between Mahsuri Mutan and BC_1_P_1_. Similarly, no significant differences were observed between Mahsuri Mutan, F_1_, F_2_, and BCP_1_ for PC. No significance differences were observed between F_1_ and F_2_ for HKE, PC, and LW. Meanwhile, between MR219 and BC_1_P_2_, there was no significant difference observed in MGW and CGW. Furthermore, there was no significant difference between F_2_ and BC_1_P1 for GCW. However, all traits showed significant differences after ageing except LW.

### 3.2. Scaling Test (MR219 × Mahsuri Mutan)

In the scaling test, the segregating generation means (BC_1_P_1_, BC_1_P_2_, and F_2_) were tested separately and called scales *A*, *B*, and *C*, respectively. The positive and negative signs in Tables 3 and 4 indicate the magnitude of gene interaction of the test scale. In the present study, the observation of the grain characteristics was based on ageing and nonageing treatments. Under nonageing treatment, CGL shows (Table 3) no significant difference for scaling *A*, *B*, and *C*. This result showed that CGL trait showed the additive-dominance model adequately for the trait under this treatment before ageing. However, the results obtained on CGL after ageing (Table 3) indicated that the entire scaling test had significant differences. The additive-dominance model cannot simply explain these trait inheritances. The trait must go through the digenic model (six-parameter model) to identify the specific gene interaction of that trait. However, Singh and Chaudhary [13] found complicating effects such as maternal effects, or gene interactions were not involved in the genetic control of these traits.

Meanwhile, MGL showed a significant difference in both treatments (Tables 3 and 4). This showed that the additive-dominance model does not adequately explain the gene interaction. Thus, further analysis through a six-parameter model should be done to identify the individual gene interaction. For MGL, most scaling tests on nonageing treatment showed significant differences except the scaling test *A* under ageing. The nonallelic interaction was shown on the entire test. Even though the scaling test *A* of this trait after ageing showed the adequacy of the additive-dominance model, it was not strong enough to explain the gene interaction of this trait. In this situation, external factors should be considered where the genotype × environment (*G* × *E*) interaction is involved. As ageing is one of the *G* × *E* factors [24], the gene expression of this trait changed after ageing. Thus, a simple additive-dominance test cannot explain such gene interactions. The same pattern was observed for MGW, HKE, PC, and LW. Slightly different patterns in CGW showed significant differences in the scaling test of both treatments. Six-parameter models can explain the gene interaction of these traits. The same results were found by Ramli et al. [10] where all the scale's components were significantly different for GL and LW. Contradictory results were reported by Ghorbanipour and Rabiei [25] who showed that the additive-dominance model was adequate for all evaluated grain quality traits, and estimation of the gene effects was performed using the three-parameter model as developed by Jinks and Jones [14]. The different results between the findings might be due to the external factor such as ageing treatment. This phenomenon happens due to the physical changes in rice grain where ageing induces changes and may give different results in milled and cooked quality of rice [26]. The results, which confirmed the adequacy of the additive-dominance model, were further partitioned into the gene effects of the evaluated traits.

### 3.3. Estimation of Gene Action and Epistasis Effects (MR219 × Mahsuri Mutan)

In the present study, scaling tests were found to be significant for most of the traits. This indicates that higher-order interaction (interallelic interaction) plays an important role in the expression of a trait, and additive-dominance alone will not be enough to deal with such traits [10]. In such conditions, available populations must be forwarded to the next generation to arrive at the best fit model [17]. There are two models used in the determination of gene effect: the three-parameter and six-parameter models explaining nonallelic interactions using the additive-dominance model test. The digenic nonallelic model with the six-parameter model [15] portrayed that the epistatic interaction model sufficiently explained the gene action in most of the traits. The six-parameter model was used to estimate the gene effect if at least one of the scales showed significance. The gene effects of each trait are summarized in Tables 5 and 6 according to the nonageing and ageing treatment, respectively. The mean effect [*m*] was significant for all studied traits in both treatments, indicating that these traits were quantitatively inherited. An additive gene effect [*d*] was significant for most traits for both treatments except PC, which showed nonsignificant difference under nonageing treatment while the value changes after ageing. This change indicates that ageing might influence the gene interaction attitude. This can be explained through the genotype × environment (*G* × *E*) interaction, where ageing is an external factor that directly influences gene interaction and expression changes, thus suggesting that the selection for these traits in the next generation could be performed in an early generation, especially in selecting high kernel elongation (HKE) trait. Therefore, the HKE trait also should be selected at the early stage. However, for PC, it is not suitable for this early selection, but it could be influenced by the high kernel elongation gene because PC is directly correlated with high kernel elongation trait for Mahsuri Mutan × MR219 crosses.

Meanwhile, for dominance [*d*], all traits were significantly different. For the interaction of additive × additive gene effect [*i*], the CGL showed no significant difference, and for the additive × dominance gene effect [*j*], there is no significant difference for traits CGL, CGW, and HKE. Then, for the dominance × dominance gene interaction, CGL, MGW, and HKE showed no significant difference with this interaction. However, under the observation, LW, MGL, and PC all had significant differences for all gene interaction models. Initially, most of the traits displayed opposite signs between additive [*d*] and dominance × dominance [*h*]. This sign showed duplicate epistasis in most of the traits except HKE, showing equal signs between both gene interactions. However, after ageing, the sign of HKE was an opposite sign which led to duplicate epistasis. Hence, in the expression of HKE, the recessive allele masked the expression of dominant alleles at two loci in order to express the HKE. Therefore, the ageing treatment induced the gene action. Therefore, for this cross, the selection of the parent should be delayed to the next generation. According to Ramli et al. [10] and Hayman [15], the classification of gene interactions depends on the magnitude and sign of the estimates of dominance [*h*] and dominance × dominance [*l*] effects. This interaction shows that there were many pairs of interacting genes. The sign associated with the estimate of additive effects [*d*] and dominance effect [*h*] indicates the parent which concentrated the highest number of genes or positive alleles for increasing the trait [10]. Therefore, the significance but positive [*d*] for HKE in both treatments indicates that the additive effect of the gene is predominant. Hence, the selection for this trait should be delayed to a later generation.

The significant and negative values of [*d*] for MGW and CGW in both treatments indicate that the inheritance of these traits is not controlled by additive gene action. Similarly, the significant and positive values of the dominance effect [*h*] for most of the traits, especially HKE, showed that the dominant effect is predominant. Thus, the selection of this trait should be delayed until heterozygosity is reduced in population. An earlier study revealed that traits with high dominance than additive could be improved through conventional breeding approaches like pedigree or bulk or single seed descent methods if selection is delayed until later generations where the dominance effect is diminished [16]. On the other hand, traits such as MGL, CGL, and PC showed significant but negative values of [*h*], [*i*], [*j*], and [*l*]. This sign indicates that negative alleles were also dispersed in the parents involved in the cross. The negative sign of [*h*] for those traits indicates that the dominance effect was having alleles responsible for low values for these traits. Therefore, the selection of such traits must also be delayed to the next generation when desirable segregates become available.

### 3.4. Generation Means of the Population Derived from MR219 × Basmati 370

The performance of the generations for the traits evaluated is presented in Table 7. The average of the F_1_ progeny for all traits is intermediate of parents. The observed values of F_1_ traits such as MGL, CGL, HKE, and LW were higher than those of MR219 and lower than those of Basmati 370. However, other traits such as MGW and CGW were higher than those of Basmati 370 and lower than those of MR219. For MGL and HKE, Basmati 370 and BC_1_P_1_ showed no differences in their mean performance. Furthermore, MR219 and BCP_2_ for MGW and CGW had no significant differences. Furthermore, no significant differences were observed between the mean performance of MR219, F_1_, and BC_1_P_1_ for PC traits. All parameters showed significant differences under both nonageing and ageing treatments.

### 3.5. Estimation of Scaling Test (MR219 × Basmati 370)

The scaling test for this population obtained under nonageing treatment was significantly different for all the traits under the three scales except for MGL which showed nonsignificant difference for scale *A* (Table 8). However, the result obtained after ageing for this trait showed that it had significant differences in all scales. Thus, the additive-dominance model was not adequate to explain the gene interaction of this trait. The same result was observed in PC where under nonageing, scale *B* was not significantly different, but after ageing, all the scales showed significant difference. However, the contrary pattern of the result was observed in HKE, where under nonageing, all scales had significant difference, but after ageing, scale *C* (Table 9) showed nonsignificant differences. The result indicated that the additive-dominance model was adequate to explain the gene interaction on HKE trait for this population. Thus, based on the additive-dominance model, the HKE trait was controlled by dominance gene interaction. However, a digenic model should be tested to confirm the gene interaction. The significant result on most traits indicated that there are epistasis effects that were similar to the previous population. The results obtained on this population also showed that the traits were influenced by higher-order (interallelic gene) interaction. Thus, the additive-dominance model was not sufficient to explain the gene interaction of these traits. The digenic model must be used to estimate the interaction of the gene individually. Based on the results, the sign of the traits was influenced by ageing treatment. The ageing caused the gene interaction to express differently, which means that it amplified the expression, especially HKE. Thus, the gene effect on HKE may be influenced by external factors like ageing. Thus, the simple additive-dominance model could not adequately estimate the gene interaction [16].

### 3.6. Estimation of Gene Action and Epistasis Effect (Basmati 370 × MR219)

Similar to the population derived from MR219 × Basmati 370, the pattern of both populations was quite similar where most traits showed significant difference in all scales in the additive-dominance model except HKE which was not significantly different in scale *C* (Tables 10 and 11). However, a previous study suggested that the additive-dominance model alone was not enough to estimate the gene interaction and explain the epistasis effect. Thus, the digenic model with the six-parameter model was used to explain gene interaction individually. Based on the result, the mean effect [*m*] was significant in all the evaluated grain quality traits for both treatments, and this suggested that all traits were quantitatively inherited. All traits under both treatments showed significant difference under additive [*d*] gene interactions. The significant difference showed that all traits are predominant. The same pattern was displayed for all traits under dominant gene interaction [*h*] for both treatments. Under additive × additive-dominance [*i*], most of the traits under nonageing showed significant result except CGW. All the traits also had significant difference after ageing treatment. The same pattern was observed in additive × dominance [*j*] and dominance × dominance [*l*] where most of the traits were significantly different under nonageing except CGL and CGW for the additive × dominance [*j*] interaction, and only CGL was not significant under the dominance × dominance [*l*] interaction. However, the pattern changed after ageing treatment where all the traits showed significant difference. Based on the observation, ageing treatment gave external effect on gene interaction where the treatment amplified the gene interaction on the traits. Thus, ageing is a factor that can modify the gene interaction especially that related to high kernel elongation. Xu et al. [24] reported that ageing has a suppression effect on grain and can improve grain expression. This might be due to the heat treatment and storage period that suppress the gene and cause the gene expression to be stronger than before ageing [26].

The pattern of epistasis effect on HKE trait under this population is contrary to the population derived from MR219 × Mahsuri Mutan where the initial result for this population (MR219 × Basmati 370) under nonageing was duplicate epistasis. However, it changed to complimentary epistasis after ageing effect. The presence of complimentary gene action for the HKE trait showed that the parents selected for crosses were diverse. It is possible to enhance genetic gain in breeding programme for improving this trait. Therefore, MR219 and Basmati 370 can be the best parent since their respective crosses showed complimentary gene action for HKE trait. As mentioned from the previous population, the estimated sign of additive [*d*] and dominance [*h*] effects showed that the associated parent has high level of gene or positive allele for increasing the trait [10]. Positive and significant additive [*d*] gene effects were observed in HKE under ageing. The same gene interaction was observed for MGL, CGL, PC, and LW. The result showed that the additive gene effect was predominant and selection for these traits should be delayed until the next generation. The negative and significant sign observed in MGW and CGW implied that the inheritance of these traits was not controlled by additive gene action.

Similarly, positive and significant dominance [*h*] gene effects were observed in HKE, MGW, CGW, PC, and LW. The result implied that dominant genes were predominant and the selection of these traits also should be delayed until heterozygosity is diminished so that negative alleles could be removed in the selection. However, the negative and significant sign for MGL and CGL showed that the dominance effect was contributed by the parents having alleles responsible for low values for these traits. The significant and same sign of [*d*] and [*h*] for HKE indicate the predominant role of additive and dominance effects for the inheritance of this trait. Hence, improvement of this trait could be achieved through recurrent selection. However, the negative and significant value of additive × additive [*i*] effect on HKE traits showed that this gene interaction did not contribute in the inheritance for this trait. The same sign was also observed for additive × dominance [*j*] gene interaction and indicated that the inheritance of such traits does not require such gene action.

### 3.7. Estimation of Heritability in the F_2_ Population

In this study, the broad sense and narrow sense heritability values range from 0.11 to 0.96 (Table 12) for all traits. For broad sense heritability, high value was observed in LW, PC, CGW, and MGM for crosses between Mahsuri Mutan × MR219 at 0.90, 0.91, 0.87, and 0.93, respectively. However, for crosses between Basmati 370 × MR219, CGL, MGW, and CGW had high broad sense heritability at 0.95, 0.85, and 0.96, respectively. For narrow sense heritability, high heritability was observed in MGL, MGW, CGW, PC, and LW for cross between Mahsuri Mutan × MR219 at 0.95, 0.93, 0.87, 0.91, and 0.90, respectively. Furthermore, CGL, MGW, and CGW had high narrow sense heritability for cross Basmati 370 × MR219 at 0.95, 0.85, and 0.96, respectively. For the trait of interest, the HKE for the cross between Mahsuri Mutan × MR219 showed 0.54 for broad sense heritability which is considered moderate heritability, while low narrow sense heritability was observed at 0.02. However, moderate broad and narrow sense heritability of 0.67 and 0.63 was observed in HKE for the cross MR219 × Basmati 370. Generally, traits with high or moderate heritability are easily passed down to the subsequent generations. The estimated heritability for high kernel elongation in crosses between Mahsuri Mutan × MR219 was lower compared to that of MR219 × Basmati 370.

### 3.8. Inheritance Pattern of High Kernel Elongation (HKE)

In the F_2_ generation, for both populations between MR219 × Mahsuri Mutan and MR219 × Basmati 370, the result showed nonsignificance difference in the chi-square test on the inheritance of normal kernel elongation to high kernel elongation with the ratio of 3 : 1. This result follows the single-gene Mendelian inheritance (Table 13), indicating that this trait is controlled by a recessive gene.

**Figure 1:**
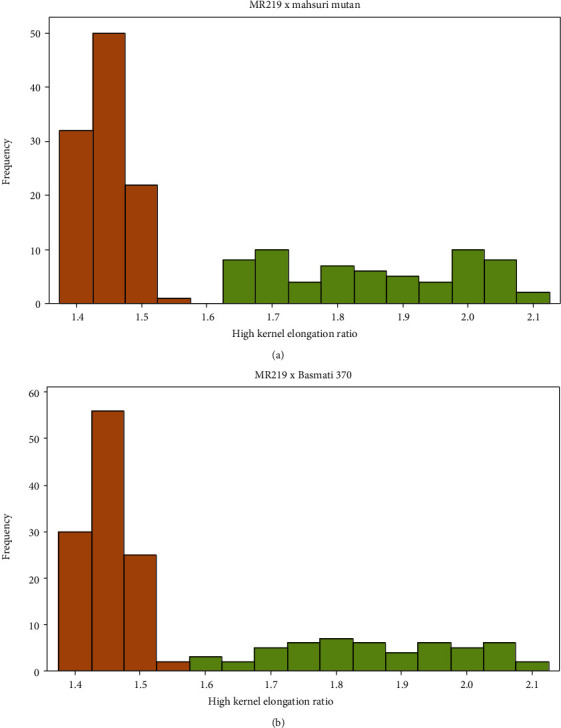
The segregation pattern of F_2_ generation of (a) MR219 × Mahsuri Mutan and (b) MR219 × Basmati 370. NKE = normal kernel elongation; HKE = high kernel elongation.

In addition, the segregation pattern also showed that the segregation followed the Mendelian ratio of 3 : 1 (Figures 1(a) and 1(b)). In both crosses, the distribution of the high kernel elongation ratio segregating populations formed a bimodal curve but skewed towards lower kernel elongation. Based on Figures 1(a) and 1(b), the best fit was achieved where HKE segregation followed 3 : 1 (nonelongation : elongation). This suggested that high kernel elongation is controlled by a recessive single gene where HKE contributed 1 in the 3 : 1 ratio of the population segregation. Similar observation was reported by Faruq et al. (2015) on the inheritance pattern of kernel elongation rice where the kernel elongation in Mahsuri and Mahsuri Mutan had similar result with the present study. The observations suggest that the kernel elongation ratio is controlled by 1 or 2 major genes and those were influenced with few modifier genes. However, the nature of kernel elongation of Mahsuri Mutan is different to that rice as high kernel elongation of the Mahsuri Mutan is only achieved after the rice ageing treatment.

### 3.9. Phenotypic Path Coefficient Analysis in High Kernel Elongation

**Figure 2:**
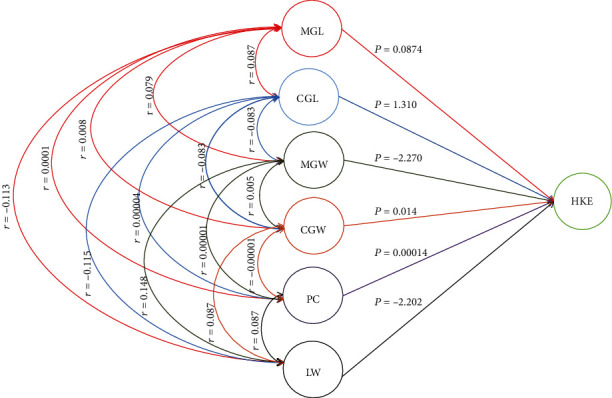
Path diagram and coefficient of factors influencing HKE. *P*_*ij*_ values are the direct effects, and *r*_*ij*_ values are the correlation coefficients. MGL: milled grain length; CGL: cooked grain length; MGW: milled grain width; CGW: cooked grain width; HKE: high kernel elongation ratio; PC: proportionate change; LW: length-width ratio.

The results obtained on direct and indirect effects of path coefficient analysis are presented in Table 14 and Figure 2. In this study, a positive significant direct effect was observed in CGL and LW at 1.31 and 0.20, respectively. However, high and moderate direct effects were observed in LW and PC through CGL at 0.40 and 0.74, respectively. On the other hand, CGL also recorded moderate negative indirect effect through MGL and MGW at -0.61 and -0.58, respectively. The negative and indirect signs showed that these characteristics were not directly related [18]. Thus, the correlation analysis did not reveal that CGL was associated with high kernel elongation characteristics. This may be due to negative indirect effect *via* MGL and MGW. Based on the analysis, the length-width ratio (LW) directly influenced the high kernel elongation character. However, there were some other traits that assisted in the expression of high kernel elongation of rice such as PC, MGL, and MGW. This result was supported by Khatun et al. [17] who found kernel length to have positive and highly significant correlation with kernel breadth after cooking but had negative and significant correlation with the kernel elongation ratio. Kernel length after cooking also had positive and highly significant relationship with the kernel elongation ratio. Hence, the ratio of changes between lengthwise and widthwise of the rice before and after cooking gave indirect effect in determining the value of HKE of rice grain. When cooking rice, kernel of rice absorbs water and increase in volume *via* increase in length and width. Lengthwise increase without an increase in girth is a desirable property in high-quality premium rice. The cooked grain length (CGL) showed the strongest indirect effect through milled grain LW to the HKE. This finding agreed with the report of Nadaf et al. [27] where kernel length expressed a positive and significant intercorrelation with the kernel LW ratio.

Cooked grain length showed the highest positive direct interaction to the high kernel elongation while LW of cooked grain exhibited negative direct relationship with the high kernel elongation ratio of rice. This could be related to the study conducted by Nadaf et al. [27] where kernel length after cooking exhibited a significant and positive association with the linear elongation ratio and water uptake, but there was a significant and negative intercorrelation with kernel breadth after cooking. This clearly indicates that selection for kernel length after cooking will improve the linear elongation ratio also, but at the same time, there will be a negative impact on kernel breadth after cooking which is desirable for the improvement of these quality traits. Therefore, when selecting for high kernel elongation, traits such as CGL, PC, and LW should be given adequate priority due to their direct and indirect path of influence in HKE. Understanding these related characters could assist in harnessing the value of high kernel elongation character for improving quality rice variety. Moreover, the knowledge of the interrelationship between grain quality and high kernel elongation traits revealed the intensity and direction of association with each other. This could facilitate effective selection for simultaneous improvement of one or more grain quality trait contributing to high kernel elongation character.

## 4. Conclusion

Ageing treatment is an external factor that could improve the expression of HKE. In general, results from the cross between Basmati 370 × MR219 showed higher value on HKE than what was obtained from Mahsuri Mutan × MR219 cross. This result revealed that the cross Basmati 370 × MR219 was superior to the Mahsuri Mutan × MR219 cross with regard to the high HKE trait. The result obtained from this study also revealed that the inheritance of the HKE trait followed Mendel's single-gene model, and it was controlled by the recessive genes. The heritability of the MR219 × Mahsuri Mutan cross was lower than that of the MR219 × Basmati 370 cross. However, there were some traits that showed high heritability values such as MGL and CGW which could be transferred to the next generation. Even though the heritability of this cross was low, the existence of high kernel elongation genes in this cross is highly prized as Mahsuri Mutan is the only local source for this gene. It was also sufficient to conclude that when selecting for high kernel elongation, traits such as CGL, PC, and LW should be given adequate priority due to their direct and indirect path of influence on HKE. This could facilitate effective selection for simultaneous improvement of one or more yield contributing traits and grain quality characters.

## Figures and Tables

**Table 1:** Analysis of variance for grain quality traits from six generations of selected populations.

Source of variance	df	Means square						
		MGL	CGL	MGW	CGW	HKE	PC	LW
*Population of cross of MR219 × Mahsuri Mutan*								
Generations (G)	5	35.82^∗∗^	900.15^∗∗^	9.08^∗∗^	7.96^∗∗^	4.47^∗∗^	4.27^∗∗^	82.41^∗∗^
Ageing (A)	1	13.01^∗∗^	846.25^∗∗^	1.13^∗∗^	15.51^∗∗^	43.26^∗∗^	0.44^∗∗^	0.015 ns
G × A	5	1.95^∗∗^	349.50^∗∗^	3.09^∗∗^	0.47^∗∗^	2.76^∗∗^	9.25^∗∗^	7.39^∗∗^
Error	24	0.104	0.188	0.478	0.595	0.692	0.442	0.151

*Population of cross of MR219 × Basmati 370*								
Generations (G)	5	819.58^∗∗^	3609.35^∗∗^	11.41^∗∗^	10.72^∗∗^	5.05^∗∗^	22.27^∗∗^	397.12^∗∗^
Ageing (A)	1	345.63^∗∗^	4742.85^∗∗^	11.33^∗∗^	87.22^∗∗^	20.27^∗∗^	75.42^∗∗^	165.61^∗∗^
G × A	5	186.65^∗∗^	673.31^∗∗^	3.32^∗∗^	0.76^∗∗^	5.42^∗∗^	39.32^∗∗^	76.34^∗∗^
Error	24	0.198	0.096	0.225	0.709	0.009	0.017	0.485

^∗∗^Significant at *P* ≤ 0.01; ^∗^significant at *P* ≤ 0.05. ns: nonsignificant difference; MGL: milled grain length; CGL: cooked grain length; MGW: milled grain width; CGW: cooked grain width; HKE: high kernel elongation ratio; PC: proportionate change; LW: length-width ratio. Population size: P_1_: 20, P_2_: 20, F_1_: 60, F_2_: 160, BCP_1_: 120, and BCP_2_: 120 for both populations.

**Table 2:** Generation means and interaction of ageing and nonageing treatments for grain quality traits in a population derived from a cross between MR219 × Mahsuri Mutan.

Traits	MGL	CGL	MGW	CGW	HKE	PC	LW
Mahsuri Mutan	6.54^a^	11.10^a^	1.76^e^	1.93^d^	1.65^a^	0.55^a^	3.71^a^
MR219	5.56^b^	7.23^e^	2.14^a^	2.38^a^	1.37^d^	0.17^c^	2.60^d^
F_1_	6.08^c^	9.72^b^	2.03^b^	2.19^b^	1.52^c^	0.49^a^	2.99^c^
F_2_	5.75^d^	9.09^c^	1.97^c^	2.13^c^	1.51^c^	0.51^a^	2.94^c^
BCP_1(Mahsuri Mutan)_	6.42^e^	11.19^a^	1.84^d^	2.11^c^	1.59^b^	0.52^a^	3.49^b^
BCP_2(MR219)_	5.44^f^	8.14^d^	2.15^a^	2.35^a^	1.37^d^	0.37^b^	2.53^e^

*Ageing treatment*							
Ageing (90°C, 3 hours)	5.83^a^	9.98^a^	2.01^a^	2.26^a^	1.62^a^	0.47^a^	3.00^a^
Nonageing	5.97^b^	8.92^b^	1.97^b^	2.11^b^	1.38^b^	0.45^b^	2.99^a^

MGL: milled grain length; CGL: cooked grain length; MGW: milled grain width; CGW: cooked grain width; HKE: high kernel elongation ratio; PC: proportionate change; LW: length-width ratio. Means followed by different letters in the same column are significantly different at *P* ≤ 0.05 based on DMRT.

**Table 3:** Scaling test of the three-parameter model for MR219 × Mahsuri Mutan cross for grain parameters under nonageing treatment.

Parameter	MGL	CGL	MGW	CGW	HKE	PC	LW
*A*	−0.292 ± 0.106^∗∗^	0.202 ± 0.348^ns^	−0.051 ± 0.015^∗∗^	−0.098 ± 0.023^∗∗^	0.037 ± 0.030^ns^	0.128 ± 0.062^∗^	−0.369 ± 0.085^ns^
*B*	0.867 ± 0.100^∗∗^	−0.299 ± 0.349^ns^	0.004 ± 0.026^ns^	−0.141 ± 0.026^∗∗^	0.086 ± 0.027^∗^	−0.171 ± 0.060^∗^	0.826 ± 0.051^∗∗^
*C*	1.691 ± 0.219^∗∗^	0.395 ± 0.660^ns^	0.486 ± 0.064^∗∗^	0.403 ± 0.060^∗∗^	0.180 ± 0.053^∗^	−1.199 ± 0.18^∗∗^	−2.526 ± 0.147^∗∗^

MGL: milled grain length; CGL: cooked grain length; MGW: milled grain width; CGW: cooked grain width; HKE: high kernel elongation ratio; PC: proportionate change; LW: length-width ratio; ns: nonsignificant. *A* = 2B_1_ − P_1_ − F_1_; *B* = 2B_2_ − P_2_ − F_1_; *C* = 4F_2_ − 2F_1_ − P_1_ − P_2_. ∗ and ∗∗ are the significant *t*-test from zero at 0.05 and 0.01 probability levels, respectively.

**Table 4:** Scaling test of the three-parameter model for MR219 × Mahsuri Mutan cross for grain parameters under ageing treatment.

Parameter	MGL	CGL	MGW	CGW	HKE	PC	LW
*A*	0.153 ± 0.112^ns^	3.368 ± 0.487^∗∗^	−0.271 ± 0.014^∗∗^	0.083 ± 0.023^∗^	0.095 ± 0.043^∗^	−0.138 ± 0.074^∗^	−0.514 ± 0.064^∗∗^
*B*	−0.654 ± 0.110^∗∗^	−1.625 ± 0.137^∗∗^	0.270 ± 0.027^∗∗^	0.127 ± 0.022^∗∗^	−0.201 ± 0.013^∗∗^	0.008 ± 0.045^ns^	0.625 ± 0.065^∗∗^
*C*	1.528 ± 0.231^∗∗^	4.207 ± 0.863^∗∗^	−0.554 ± 0.061^∗∗^	−0.437 ± 0.078^∗∗^	−0.304 ± 0.076^∗∗^	0.557 ± 0.113^∗∗^	−0.317 ± 0.103^∗∗^

MGL: milled grain length; CGL: cooked grain length; MGW: milled grain width; CGW: cooked grain width; HKE: high kernel elongation ratio; PC: proportionate change; LW: length-width ratio; ns: nonsignificant. *A* = 2B_1_ − P_1_ − F_1_; *B* = 2B_2_ − P_2_ − F_1_; *C* = 4F_2_ − 2F_1_ − P_1_ − P_2_. ∗ and ∗∗ are the significant *t*-test from zero at 0.05 and 0.01 probability levels, respectively.

**Table 5:** Estimation of gene effect under nonageing in MR219 × Mahsuri Mutan cross.

Character	Means (*m*)	Additive (*d*)	Dominance (*h*)	Additive × additive (*i*)	Additive × dominant (*j*)	Dominant × dominant (*l*)	Type of epistasis
MGL	5.746 ± 0.042^∗∗^	0.999 ± 0.047^∗∗^	1.229 ± 0.205^∗∗^	1.116 ± 0.193^∗∗^	1.160 ± 0.118^∗∗^	−0.541 ± 0.288^∗∗^	Duplicate
CGL	8.815 ± 0.113^∗∗^	1.044 ± 0.149^∗∗^	1.721 ± 0.593^∗^	0.492 ± 0.542^ns^	−0.500 ± 0.406^ns^	−0.589 ± 0.890^ns^	Duplicate
MGW	1.851 ± 0.015^∗∗^	−0.173 ± 0.008^∗∗^	0.590 ± 0.062^∗^	0.533 ± 0.060^∗^	0.055 ± 0.028^∗∗^	−0.579 ± 0.072^ns^	Duplicate
CGW	2.019 ± 0.013^∗∗^	−0.249 ± 0.009^∗∗^	0.675 ± 0.057^∗∗^	0.643 ± 0.054^∗^	−0.022 ± 0.015^ns^	−0.882 ± 0.071^∗∗^	Duplicate
HKE	1.321 ± 0.008^∗∗^	0.074 ± 0.009^∗∗^	0.111 ± 0.043^∗^	0.055 ± 0.038^∗^	0.052 ± 0.039^ns^	0.070 ± 0.064^ns^	Complimentary
PC	0.649 ± 0.040^∗∗^	−0.029 ± 0.026^ns^	−0.962 ± 0.173^∗∗^	−1.156 ± 0.168^∗∗^	−0.299 ± 0.071^∗∗^	1.113 ± 0.208^∗∗^	Duplicate
LW	2.813 ± 0.037^∗∗^	0.770 ± 0.027^∗∗^	0.877 ± 0.163^∗∗^	0.968 ± 0.158^∗∗^	0.454 ± 0.075^∗∗^	−0.559 ± 0.2^∗∗^	Duplicate

∗ and ∗∗ are significant to the *χ*^2^ test, from 0 to 0.5 probability. *χ*^2^ tabulated value = 7.81. ns: nonsignificant difference; MGL: milled grain length; CGL: cooked grain length; MGW: milled grain width; GCW: cooked grain width; HKE: high kernel elongation ratio; PC: proportionate change; LW: length-width ratio.

**Table 6:** Estimation of gene effect under ageing in MR219 × Mahsuri Mutan cross.

Character	Means (*m*)	Additive (*d*)	Dominant (*h*)	Additive × additive (*i*)	Additive × dominant (*j*)	Dominant × dominant (*l*)	Type of epistasis
MGL	6.342 ± 0.045^∗∗^	0.962 ± 0.041^∗∗^	−2.074 ± 0.211^∗∗^	−2.028 ± 0.198^∗∗^	0.404 ± 0.074^∗∗^	2.529 ± 0.283^∗∗^	Duplicate
CGL	11.018 ± 0.185^∗∗^	5.071 ± 0.143^∗∗^	−2.588 ± 0.823^∗∗^	−2.465 ± 0.792^∗∗^	2.497 ± 0.240^∗∗^	0.722 ± 1.036^ns^	Duplicate
MGW	1.875 ± 0.013^∗∗^	−0.452 ± 0.009^∗∗^	0.664 ± 0.057^∗∗^	0.553 ± 0.055^∗∗^	−0.271 ± 0.013^∗∗^	−0.552 ± 0.070^∗∗^	Duplicate
CGW	2.121 ± 0.018^∗∗^	−0.243 ± 0.009^∗∗^	0.680 ± 0.076^∗∗^	0.647 ± 0.074^∗∗^	−0.022 ± 0.014^ns^	−0.857 ± 0.087^∗∗^	Duplicate
HKE	1.580 ± 0.011^∗^	0.361 ± 0.007^∗^	0.176 ± 0.049^∗^	0.197 ± 0.047^∗^	0.296 ± 0.030^∗^	−0.091 ± 0.061^ns^	Duplicate
PC	0.366 ± 0.020^∗∗^	0.326 ± 0.025^∗∗^	0.762 ± 0.102^∗∗^	0.688 ± 0.094^∗∗^	0.147 ± 0.079^∗∗^	−0.818 ± 0.15^ns^	Duplicate
LW	3.073 ± 0.015^∗∗^	1.140 ± 0.024^∗∗^	−0.664 ± 0.086^∗∗^	−0.428 ± 0.075^∗∗^	1.139 ± 0.085^∗∗^	0.539 ± 0.140^∗∗^	Duplicate

∗ and ∗∗ are significant to the *χ*^2^ test, from 0 to 0.5 probability. *χ*^2^ tabulated value = 7. ns: nonsignificant difference; MGL: milled grain length; CGL: cooked grain length; MGW: milled grain width; GCW: cooked grain width; HKE: high kernel elongation ratio; PC: proportionate change; LW: length-width ratio.

**Table 7:** Generation means and interaction of ageing and nonageing treatments for grain quality traits in a population derived from a cross between MR219 × Basmati 370.

Traits	MGL	CGL	MGW	CGW	HKE	PC	LW
Basmati 370	8.48^a^	15.40^a^	1.76^e^	1.99^e^	1.66^a^	0.21^a^	4.84^a^
MR219	5.20^e^	7.24^b^	2.20^a^	2.44^a^	1.45^d^	0.02^c^	2.37^b^
F_1_	6.86^c^	11.02^c^	2.01^c^	2.23^b^	1.59^c^	0.03^bc^	3.41^c^
F_2_	7.18^b^	9.85^d^	2.08^b^	2.19^c^	1.51^b^	0.08^d^	3.53^d^
BCP_1_	8.47^a^	14.16^e^	1.85^d^	2.12^d^	1.67^a^	0.23^c^	4.59^e^
BCP_2_	5.39^d^	8.17^f^	2.19^a^	2.42^a^	1.49^c^	0.09^b^	2.47^f^
*Ageing treatment*							
Ageing (90°C, 3 hours)	6.66^b^	11.99^a^	2.09^a^	2.41^a^	1.64^a^	0.17^a^	3.76^a^
Nonageing	7.33^a^	9.48^b^	1.97^b^	2.07^b^	1.47^b^	0.15^b^	3.29^b^

MGL: milled grain length; CGL: cooked grain length; MGW: milled grain width; CGW: cooked grain width; HKE: high kernel elongation ratio; PC: proportionate change; LW: length-width ratio. Means followed by different letters in the same column are significantly different at *P* ≤ 0.05 based on DMRT.

**Table 8:** Scaling test of MR219 × Basmati 370 cross for grain parameter under ageing treatment.

Parameter	MGL	CGL	MGW	CGW	HKE	PC	LW
*A*	0.153 ± 0.112^ns^	3.368 ± 0.487^∗∗^	−0.271 ± 0.014^∗∗^	0.083 ± 0.023^∗^	0.095 ± 0.043^∗^	−0.138 ± 0.074^∗^	−0.514 ± 0.064^∗∗^
*B*	−0.654 ± 0.110^∗∗^	−1.625 ± 0.137^∗∗^	0.270 ± 0.027^∗∗^	0.127 ± 0.022^∗∗^	−0.201 ± 0.013^∗∗^	0.008 ± 0.045 ns	0.625 ± 0.065^∗∗^
*C*	1.528 ± 0.231^∗∗^	4.207 ± 0.863^∗∗^	−0.554 ± 0.061^∗∗^	−0.437 ± 0.078^∗∗^	−0.304 ± 0.076^∗∗^	0.557 ± 0.113^∗∗^	−0.317 ± 0.103^∗∗^

MGL: milled grain length; CGL: cooked grain length; MGW: milled grain width; CGW: cooked grain width; HKE: high kernel elongation ratio; PC: proportionate change; LW: length-width ratio; ns: nonsignificant. *A* = 2B_1_ − P_1_ − F_1_; *B* = 2B_2_ − P_2_ − F_1_; *C* = 4F_2_ − 2F_1_ − P_1_ − P_2_. ∗ and ∗∗ are the significant *t*-test from zero at 0.05 and 0.01 probability levels, respectively.

**Table 9:** Scaling test of Basmati 370 × MR219 cross for grain parameter under nonageing treatment.

Parameter	MGL	CGL	MGW	CGW	HKE	PC	LW
*A*	−1.340 ± 0.090^∗∗^	1.340 ± 0.090^∗∗^	0.126 ± 0.029^∗∗^	0.041 ± 0.022^∗^	0.094 ± 0.019^∗∗^	−0.179 ± 0.019^∗∗^	3.788 ± 0.025^∗∗^
*B*	1.678 ± 0.082^∗∗^	−1.678 ± 0.082^∗∗^	−0.031 ± 0.027^ns^	0.028 ± 0.027^ns^	−0.354 ± 0.015^∗∗^	−0.026 ± 0.018^∗∗^	−1.563 ± 0.022^∗∗^
*C*	−5.546 ± 0.144^∗∗^	5.546 ± 0.144^∗∗^	−0.670 ± 0.064^∗∗^	−0.176 ± 0.090^∗^	−0.182 ± 0.037^ns^	0.027 ± 0.032^∗∗^	−0.352 ± 0.040^∗∗^

MGL: milled grain length; CGL: cooked grain length; MGW: milled grain width; CGW: cooked grain width; HKE: high kernel elongation ratio; PC: proportionate change; LW: length-width ratio; ns: nonsignificant. *A* = 2B_1_ − P_1_ − F_1_; *B* = 2B_2_ − P_2_ − F_1_; *C* = 4F_2_ − 2F_1_ − P_1_ − P_2_. ∗ and ∗∗ are the significant *t*-test from zero at 0.05 and 0.01 probability levels, respectively.

**Table 10:** Estimation of gene effect under nonageing in MR219 × Basmati 370 cross.

Character	Means (*m*)	Additive (*d*)	Dominant (*h*)	Additive × additive (*i*)	Additive × dominant (*j*)	Dominant × dominant (*l*)	Type of epistasis
MGL	8.317 ± 0.024^∗∗^	2.963 ± 0.037^∗∗^	−5.878 ± 0.133^∗∗^	−5.884 ± 0.122^∗∗^	1.509 ± 0.055^∗∗^	6.222 ± 0.208^∗∗^	Duplicate
CGL	11.918 ± 0.238^∗∗^	3.204 ± 0.127^∗∗^	−7.213 ± 1.002^∗^	−6.891 ± 0.983^ns^	−0.108 ± 0.197^ns^	6.517 ± 1.143^ns^	Duplicate
MGW	1.791 ± 0.014^∗∗^	−0.146 ± 0.016^∗∗^	0.772 ± 0.063^∗∗^	0.765 ± 0.061^∗∗^	0.078 ± 0.018^∗∗^	−0.860 ± 0.084^∗∗^	Duplicate
CGW	2.012 ± 0.021^∗∗^	−0.265 ± 0.011^∗∗^	0.255 ± 0.089^∗^	0.246 ± 0.087^∗^	0.007 ± 0.016^ns^	−0.315 ± 0.100^∗∗^	Duplicate
HKE	1.444 ± 0.006^∗∗^	−0.076 ± 0.008^∗∗^	0.215 ± 0.031^∗∗^	0.196 ± 0.029^∗∗^	−0.453 ± 0.021^∗∗^	−0.457 ± 0.045^∗∗^	Duplicate
PC	−0.592 ± 0.014^∗∗^	−0.308 ± 0.025^∗∗^	2.606 ± 0.083^∗∗^	2.692 ± 0.076^∗∗^	−0.935 ± 0.075^∗∗^	−2.998 ± 0.133^∗∗^	Duplicate
LW	4.242 ± 0.030^∗∗^	1.765 ± 0.033^∗∗^	−3.123 ± 0.144^∗∗^	−2.983 ± 0.138^∗∗^	1.195 ± 0.092^∗∗^	3.440 ± 0.198^∗^	Duplicate

∗ and ∗∗ are significant to the *χ*^2^ test, from 0 to 0.5 probability. *χ*^2^ tabulated value = 7.81. ns: nonsignificant difference; MGL: milled grain length; CGL: cooked grain length; MGW: milled grain width; CGW: cooked grain width; HKE: high kernel elongation ratio; PC: proportionate change; LW: length-width ratio.

**Table 11:** Estimation of gene effect under ageing in MR219 × Basmati 370 cross.

Character	Means (*m*)	Additive (*d*)	Dominant (*h*)	Additive × additive (*i*)	Additive × dominant (*j*)	Dominant × dominant (*l*)	Type of epistasis
MGL	7.633 ± 0.105^∗∗^	3.067 ± 0.065^∗∗^	−2.402 ± 0.468^∗∗^	−2.357 ± 0.439^∗∗^	1.597 ± 0.164^∗∗^	1.634 ± 0.588^∗^	Duplicate
CGL	14.203 ± 0.338^∗∗^	8.779 ± 0.207^∗∗^	−8.569 ± 1.452^∗∗^	−8.279 ± 1.413^∗∗^	3.937 ± 0.353^∗∗^	8.720 ± 1.720^∗∗^	Duplicate
MGW	1.912 ± 0.019^∗∗^	−0.539 ± 0.009^∗∗^	0.6760.079^∗∗^	0.616 ± 0.078^∗∗^	−0.314 ± 0.014^∗∗^	−0.746 ± 0.089^∗∗^	Duplicate
CGW	2.322 ± 0.013^∗∗^	−0.334 ± 0.009^∗∗^	0.622 ± 0.057^∗∗^	0.601 ± 0.055^∗∗^	−0.159 ± 0.015^∗∗^	−0.924 ± 0.069^∗∗^	Duplicate
HKE	1.638 ± 0.013^∗^	0.210 ± 0.022^∗^	0.748 ± 0.076^∗^	−0.289 ± 0.067^∗^	−0.358 ± 0.070^∗^	1.210 ± 0.123^∗^	Complimentary
PC	0.426 ± 0.028^∗∗^	0.132 ± 0.024^∗∗^	−1.729 ± 0.129^∗∗^	−1.679 ± 0.123^∗∗^	0.170 ± 0.076^∗∗^	2.213 ± 0.167^∗∗^	Duplicate
LW	2.810 ± 0.048^∗∗^	2.469 ± 0.022^∗∗^	2.774 ± 0.201^∗∗^	3.016 ± 0.198^∗∗^	2.329 ± 0.065^∗∗^	−3.670 ± 0.221^∗∗^	Duplicate

∗ and ∗∗ are significant to the *χ*^2^ test, from 0 to 0.5 probability. *χ*^2^ tabulated value = 7.81. ns: nonsignificant difference; MGL: milled grain length; CGL: cooked grain length; MGW: milled grain width; CGW: cooked grain width; HKE: high kernel elongation ratio; PC: proportionate change; LW: length-width ratio.

**Table 12:** Heritability of grain quality traits in the F_2_ populations.

Traits	Mahsuri Mutan × MR219		Basmati 370 × MR219	
	*h* ^2^_b_	*h* ^2^_n_	*h* ^2^_b_	*h* ^2^_n_
MGL	0.74	0.95	0.52	0.11
CGL	0.57	0.42	0.95	0.95
MGW	0.93	0.93	0.85	0.85
CGW	0.87	0.87	0.96	0.96
HKE	0.54	0.02	0.67	0.63
PC	0.91	0.91	0.53	0.41
LW	0.90	0.90	0.33	0.51

MGL: milled grain length; CGL: cooked grain length; MGW: milled grain width; CGW: cooked grain width; HKE: high kernel elongation ratio; PC: proportionate change; LW: length-width ratio.

**Table 13:** Inheritance pattern of HKE for MR219 × Mahsuri Mutan and MR219 × Basmati 370.

Cross	Observation on segregation		Expected ratio	*χ* ^2^	*P*
	NE	HKE			
MR219 × Mahsuri Mutan	110	50	3 : 01	3.3 ns	0.068
MR219 × Basmati 370	114	46	3 : 01	1.21 ns	0.273

Note: ^∗^*P* ≤ 0.05 and ^∗∗^*P* ≤ 0.01. ns = nonsignificant; *χ*^2^ = chi-square; NE = normal elongation; HKE = high kernel elongation.

**Table 14:** Phenotypic path analysis of the direct effect levels among six traits associated with HKE of rice.

Variable	MGL	CGL	MGW	CGW	PC	LW
MGL	0.08748^ns^	0.08748	-0.08275	-0.05186	-0.09517	0.16695
CGL	-0.61138	1.31001^∗∗^	-0.58361	0.02410	0.40113	0.74107
MGW	0.07901	0.12034	-0.27013^∗∗^	-0.09247	-0.01737	0.19625
CGW	-0.00775	0.00027	0.00494	0.01443^ns^	-0.00121	-0.00469
PC	0.00013	0.00004	0.00001	-0.00001	0.00014^ns^	-0.00006
LW	-0.11258	-0.1146	0.14729	0.06587	0.08685	0.20274^∗∗^

Note: ^∗^*P* ≤ 0.05 and ^∗∗^*P* ≤ 0.01. ns: nonsignificance; MGL: milled grain length; CGL: cooked grain length; MGW: milled grain width; CGW: cooked grain width; HKE: high kernel elongation ratio; PC: proportionate change; LW: length-width ratio.

## Data Availability

All data used to derive the result of this study were included within the tables and figures.
